# Simultaneous Detection of Parasitic Vector Borne Diseases: A Robust Cross-Sectional Survey in Hunting, Stray and Sheep Dogs in a Mediterranean Area

**DOI:** 10.3389/fvets.2019.00288

**Published:** 2019-08-29

**Authors:** Manuela Gizzarelli, Valentina Foglia Manzillo, Lavinia Ciuca, Maria Elena Morgoglione, Nour El Houda Ben Fayala, Giuseppe Cringoli, Gaetano Oliva, Laura Rinaldi, Maria Paola Maurelli

**Affiliations:** ^1^Department of Veterinary Medicine and Animal Production, University of Naples Federico II, Naples, Italy; ^2^CREMOPAR Campania Region, Naples, Italy

**Keywords:** canine vector borne diseases (CVBDs), *Leishmania infantum*, *Dirofilaria immitis*, *Dirofilaria repens*, *Acanthocheilonema reconditum*, *Babesia* spp., dogs, Italy

## Abstract

Canine vector-borne diseases (CVBDs) are a spectrum of diseases caused by different pathogens transmitted by blood-feeding arthropoda. The aim of this study was to investigate leishmaniosis, babesiosis, and filarial infections in dogs with three different lifestyles (hunting, stray, and sheep dogs) in Molise, the smallest region of southern Italy, where data available about these parasitic infections are very scant. A cross-sectional survey was conducted on 318 hunting, 180 stray, and 218 sheep dogs. Immunofluorescence antibody test, blood smear, molecular techniques and Knott's test were performed to detect *Leishmania infantum, Babesia* spp. and filarial nematodes. Association between positivity to CVBDs, age, sex, and living conditions was evaluated. An overall prevalence of 12.3% of CVBDs caused by *L. infantum* (10.2%), *B. canis canis* (0.3%) and filarial nematodes (2.1%) was detected. Three dogs showed co-infections of *L. infantum* and *B. c. canis* (0.1%) or *Acanthocheilonema reconditum* (0.3%). A significantly association was found only for filarial infection in hunting dogs. These parasites were reported also in dogs without clinical signs. It is very important to plan effective control programs for CVBDs to guarantee not only the health and welfare of pets, but also the public safety, because some of mentioned parasites are of zoonotic importance.

## Introduction

Canine vector-borne diseases (CVBDs) are a spectrum of diseases caused by different infectious/parasitic pathogens transmitted by blood-feeding arthropoda such as fleas, lice, mosquitoes, phlebotomine sand flies and ticks (1). The most common CBVDs are anaplasmosis, babesiosis, bartonellosis, borreliosis, dirofilariosis, ehrlichiosis, leishmaniosis, rickettsiosis, dipylidosis, and thelaziosis (2). Most of these CVBDs are important not only for animal health and welfare, but also because they are of major zoonotic concern (e.g., *Babesia venatorum, Babesia microti, Dirofilaria immitis, Dirofilaria repens, Leishmania* spp.) (3). The interest on CVBDs has grown in the last two decades and therefore an increased number of studies have been published in the recent few years (4). The epidemiology of CVBDs (i.e., geographical distribution, prevalence, and pathogenicity) is changing due to several factors, especially climatic changes, ecosystem changes, increased mobility of dogs and humans and developing phenomena of chemoresistance to insecticides and acaricides (5). Consequently, CVBDs are spreading into areas considered non-endemic until recently (4).

Often, CVBDs cause chronic and asymptomatic infections and their diagnosis requires specific tests (6). Furthermore, co-infections are common, especially in areas suitable for many vector species, thus changing clinical manifestations and complicating diagnosis, therapy and prognosis (7–9). If dogs are not properly treated for CVBDs, they may act as reservoir of them, representing a zoonotic risk especially as the consequence of the increasing phenomenon of cohabitation with humans in urban and rural environments (10–13). Therefore, diagnosis and control of CVBDs are highly complex and challenging (5).

The epidemiological scenario of canine and feline vector-borne diseases in Italy has been recently reviewed (14). While most of regions have been investigated for CVBDs, a dearth of data are present for some regions of central-southern Italy. The aim of the present study was to investigate three parasitic CVBDs (leishmaniosis, babesiosis and filarial infections) in dogs with three different life styles (hunting, stray and sheep dogs) in Molise, the smallest region of southern Italy where data available about these parasitic infections are very scant.

## Materials and Methods

### Study Area and Collection of Samples

A cross-sectional survey was conducted between June 2017 and June 2018 in the Molise region of southern Italy (Latitude = 41°40′00″N; Longitude = 14°30′00″E) which extends over an area of 4,438 km^2^. The region is mainly mountainous and extends from 0 to 2,185 m above sea level. The climate is cold-temperate in the western part and Mediterranean in the eastern part of the region.

**Figure 1:**
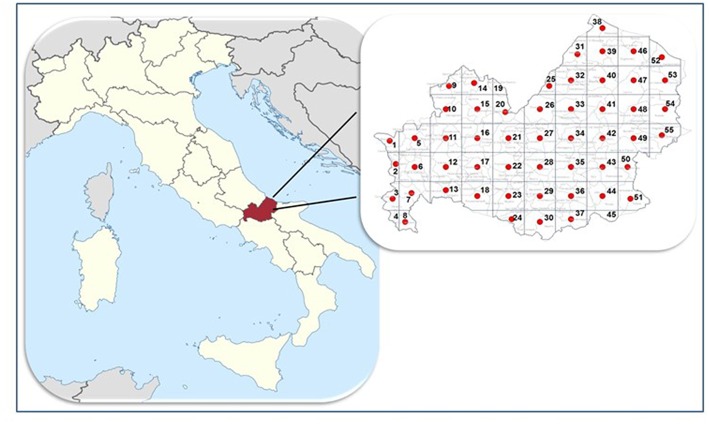
Study area. Molise region, Italy.

A grid-based approach within a Geographical Information System (GIS) was used in order to uniformly sample the dogs throughout the entire region (15). For this purpose, a grid representing quadrants of 10 × 10 km was overlaid on the regional map within the GIS. Thus, the Molise region was divided into 55 quadrants and the study was designed to sample 6 hunting, 6 stray and 6 sheep dogs in each quadrant (Figure 1) for a total of 990 dogs. From each dog, blood samples were collected as follows: 2 ml in tubes with EDTA and 2 ml in tubes with serum separator gel. Serum was separated by centrifugation at 360 g for 15 min and stored at −20°C until analysis.

All applicable international, national and/or institutional guidelines for the care and use of animals were followed. Data on age, sex and living conditions (hunting, stray, sheep dogs) were registered. Moreover, dogs were submitted for physical examination and a clinical form was completed.

### Detection of Antibodies to *Leishmania infantum*

Serum samples were analyzed by an immunofluorescence antibody test (IFAT) provided by the National Reference Center for Leishmaniosis (CReNaL, Palermo, Italy) to detect anti-*Leishmania* antibodies (sensitivity = 96% and a specificity = 98%). Antigens used by CReNaL were promastigotes of strain MHOM/TN/80/IPT1. Samples were considered positive, if they showed a titer ≥1:160 (16). Reading was performed using a fluorescence microscope (Leica DM 2500, Germany) by three independent technicians.

### Detection of *Babesia*

Blood smears were prepared using blood samples in EDTA and stained using Differential Quick Stain kit (Modified Giemsa) (Polysciences, Inc. Warrington, PA, USA), according to the manufacturer's instructions for detection of *Babesia* protozoa. Samples were analyzed by an expert examiner. Positive samples were then analyzed by molecular techniques. DNA was extracted by blood samples using the DNeasy Blood & Tissue Kit (QIAGEN, Germany). A semi-nested Polymerase chain reaction (PCR) was performed to amplify the 18S ribosomal RNA gene (17). The PCR products were detected on 2% ethidium bromide-stained low melting agarose gel (BIO-RAD, Spain). Positive amplicons were purified by QIAquick PCR purification kit (QIAGEN, Germany). The purified PCR products were sequenced in both forward and reverse directions and were analyzed by the Chromas version 2.1 software and finally compared with the NCBI/GenBank database using the Basic Local Alignment Search Tool (BLAST) and ClustalW software.

### Detection of Microfilariae

Blood samples in EDTA were analyzed by the modified Knott's test to detect microfilariae of *Dirofilaria immitis, D. repens* and *Acanthocheilonema reconditum* (18, 19). The circulating microfilariae (mf) were identified based on their morphology and morphometry (19) and counted (mf/ml of blood) in 20 ul of blood. Morphometric analyses of the mf were then performed with a standard microscope equipped with calibrated measuring eyepieces at final magnification of 200–400×. Body length and diameter of 10 randomly selected mf were determined.

### Statistical Analysis

Chi-squared test was performed using SPSS 23.0 software (IBM, Armonk, NY, USA) to study the association between positivity to CVBDs and dog's characteristics (age, sex, living conditions). Differences were considered significant at *P* < 0.05.

In addition, a spatial analysis was conducted to detect possible clusters of positive samples, using the average nearest neighbor (ANNI) index (20). If the index is < 1, the pattern exhibits clustering, while index of > 1 indicates a trend toward dispersion. A significance level of 99% was chosen in the analysis. Moreover, to confirm results obtained, the Moran's I test was used (21). A low negative z-score indicates a statistically significant spatial data outlier. A significance level of 95% was chosen for this test.

## Results

### Detection of CVBDs

A total of 716 blood and serum samples (72.3% of the expected sample) were collected: 318 from hunting, 180 from stray, and 218 from sheep dogs.

The age of animals ranged from 3 months to 17 years (median age = 3 years). Based on their age, dogs were divided into 5 classes: (a) 3–12 months; (b) 13–36 months; (c) 37–72 months; (d) 73–120 months; (e) 121–204 months. Regarding sex, 328/716 dogs were females (45.8, 95%, CI = 42.1–49.5%) and 388 males (54.2%, 95% CI = 50.5–57.9%).

An overall prevalence of 12.3% (95% CI = 10.0–15.0%) was detected for CVBDs caused by *Leishmania infantum, Babesia* spp. or filarial nematodes.

**Figure 2:**
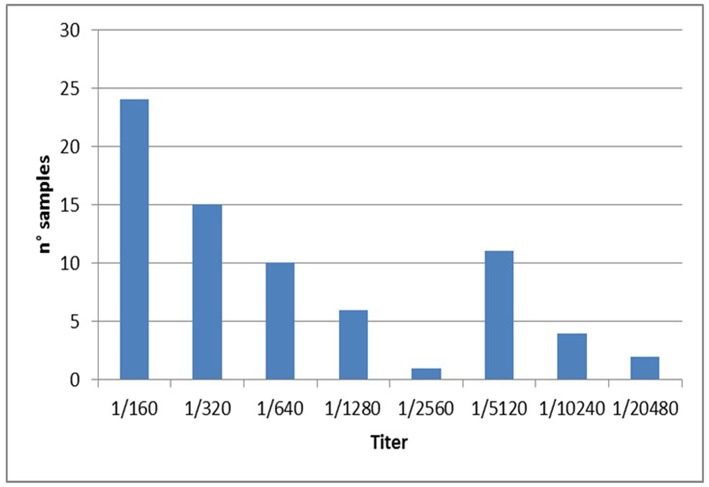
No. of dogs positive to *Leishmania* distributed by antibody titres.

Specifically, a total of 73 dogs resulted positive for leishmaniosis (10.2%, 95% CI = 8.1–12.7%) with titers ≥ 1:160 up to 1:20480 (Figure 2; Tables 1–3). The positivity was not significantly associated with age, sex and living conditions (*P* > 0.05).

**Figure 3:**
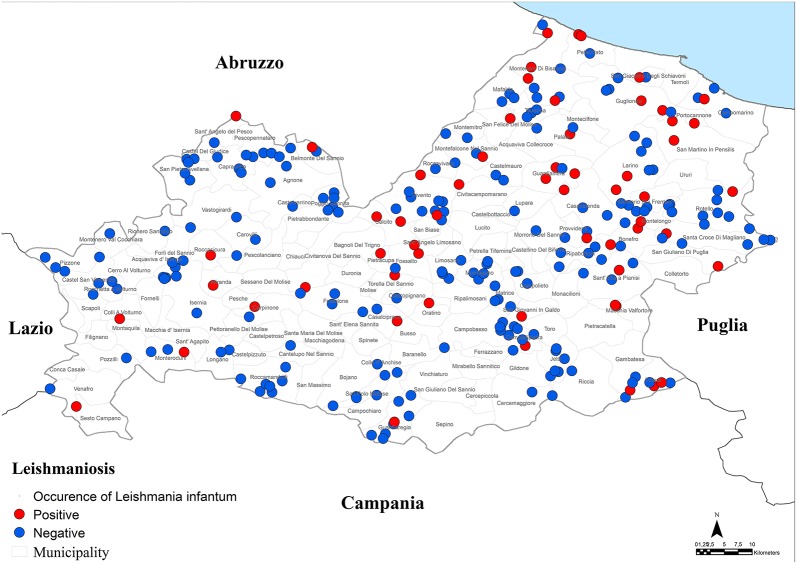
Distribution of dogs positive to *Leishmania infantum*.

**Table 1:** Prevalence and 95% CI of positive samples to *Leishmania infantum, Babesia canis canis* and filarial infection for each class of age tested.

**Class**	**Age of dogs (months)**	**No. samples analyzed**	***Leishmania infantum***		***Babesia canis canis***		**Filarial infection**				
			**No. samples with *Leishmania*-IFAT titer ≥ 1:160**	**Prevalence % (95% CI)**	**No positive samples**	**Prevalence %(95% CI)**	***D. immitis***	***A. reconditum***	***D. repens***	**Co-infection**	**Prevalence% (95% CI)**
A	3–12	146	7	4.8 (2.1–10.0)	0	0	0	2	1	0	2.1 (0.5–6.4)
B	13–36	238	29	12.2 (8.4–17.2)	1	0.4 (0.02–2.7)	1	2	2	1	1.7 (0.5–4.5)
C	37–72	194	20	10.3 (6.6–15.7)	1	0.5 (0.03–3.3)	1	3	1	1	2.1 (0.7–5.5)
D	73–120	110	12	10.9 (6.0–18.6)	0	0	0	3	2	1	3.7 (1.2–9.6)
E	121–204	28	5	17.9 (6.8–37.6)	0	0	0	0	0	0	0
	Total positive samples	716	73	10.2 (8.1–12.7)	2	0.3 (0.1–1.1)	2	10	6	3	2.1 (1.2–3.5)

**Table 2:** Prevalence and 95% CI of positive samples to *Leishmania infantum, Babesia canis canis* and filarial infection for sex.

**Sex**	**No. samples analyzed**	***Leishmania infantum***		***Babesia canis canis***		**Filarial infection**				
		**No. samples with *Leishmania*-IFAT titer ≥ 1:160**	**Prevalence % (95% CI)**	***No. positive samples***	**Prevalence % (95% CI)**	***D. immitis***	***A. reconditum***	***D. repens***	**Co-infection**	**Prevalence% (95% CI)**
Male	388	42	10.8 (8.0–14.5)	1	0.3 (0.01–1.7)	0	6	3	0	2.3 (1.1–4.5)
Female	328	31	9.5 (6.6–13.3)	1	0.3 (0.02– 2.0)	2	4	3	3	1.8 (0.8–4.1)
Total positive samples	716	73	10.2 (8.1–12.7)	2	0.3 (0.05–1.1)	2	10	6	3	2.1 (1.2–3.5)

**Table 3:** Prevalence and 95% CI of positive samples to *Leishmania infantum, Babesia canis canis* and filarial infection for each living condition tested (hunting, stray and sheep dogs).

**Living condition of dogs**	**No. samples analyzed**	***Leishmania infantum***		***Babesia canis canis***		**Filarial infection**				
		**No. samples with *Leishmania*-IFAT titer ≥ 1:160**	**Prevalence % (95% CI)**	**No. positive samples**	**Prevalence % (95% CI)**	***D. immitis***	***A. reconditum***	***D. repens***	**Co-infection**	**Prevalence % (95% CI)**
Hunting	318	29	9.1 (6.3–13.0)	2	0.3% (0.1–1.1%)	2	8	5	3	3.8 (2.1–6.7)^*^
Stray	180	23	12.8 (8.4–18.8)	0	0	0	2	1	0	1.7 (0.4–5.2)
Sheep	218	21	9.6 (6.2–14.5)	0	0	0	0	0	0	0
Total positive samples	716	73	10.2 (8.1–12.7)	2	0.3% (0.1–1.1%)	2	10	6	3	2.1 (1.2–3.5)

* *Significantly different χ2 = 8.2; P = 0.017*.

**Figure 4:**
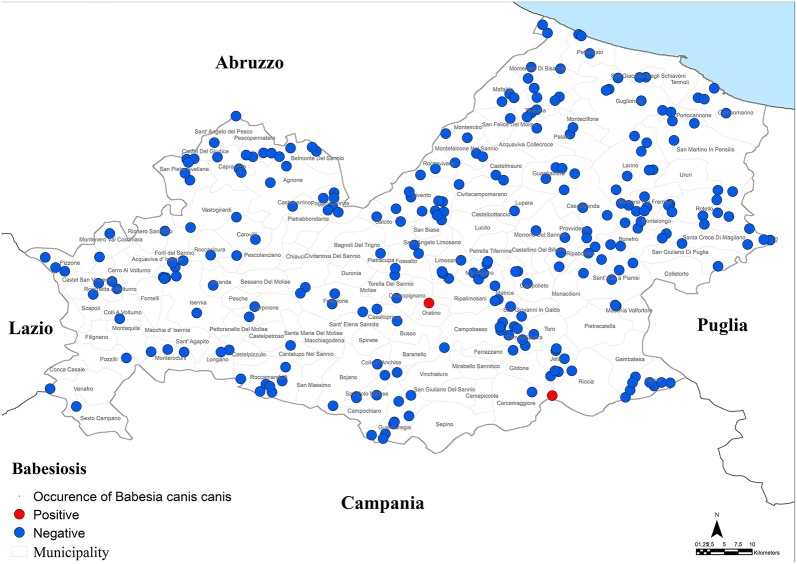
Distribution of dogs positive to *Babesia canis canis*.

Results of microscopic analysis of blood smear for *Babesia* spp. showed that 2 dogs had large intraerythrocytic piroplasms compatible with a large *Babesia*. These samples were analyzed also by PCR and sequencing and an identity of 100% with *B. canis canis* sequence (GenBank accession number KX236456.1) was found. The two positive dogs for *B. canis canis* (0.3%, 95% CI = 0.1–1.1%) were both hunting dogs: 1 male of 40 months old and 1 female of 36 months old (Figure 4; Tables 1–3).

**Figure 5:**
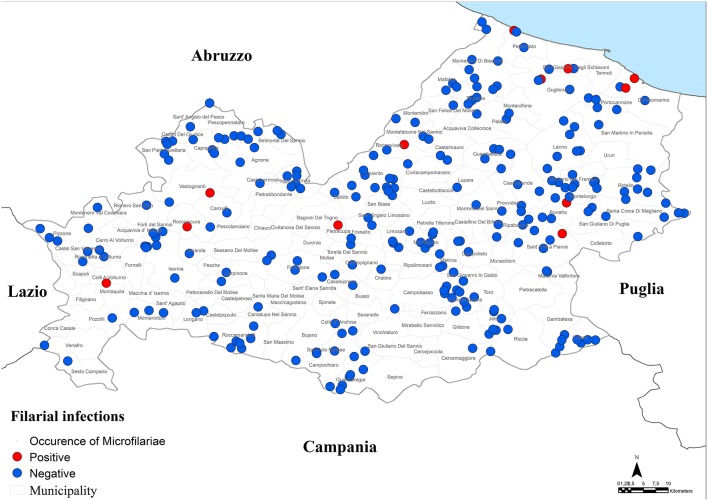
Distribution of dogs positive to filarial infections.

Moreover, a total of 15 dogs (2.1%, 95% CI = 1.2–3.5%) resulted positive for filarial infections (Figure 5). Specifically, 10 samples (1.3%; 95% CI = 0.7–2.5) were positive for *A. reconditum*, 6 (0.8%; 95% CI = 0.3–1.9) for *D. repens* and 2 (0.3%; 95% CI = 0.1–1.1) for *D. immitis*, with a higher significantly associated prevalence (*P* = 0.017) in hunting dogs (3.8%; 95% CI = 2.1–6.7), but positivity was not associated with age and sex (*P* > 0.05) (Tables 1–3). Two samples were co-infected with *A. reconditum* and *D. immitis*, while 1 sample with *A. reconditum* and *D. repens*. Finally, 2 samples showed co-infections of *L. infantum* and *A. reconditum*, whilst 1 sample showed a co-infection of *L. infantum* and *B. canis canis*.

### Clinical Signs

Only 37% (95% CI = 26.2–49.1%) of positive dogs showed clinical signs for leishmaniosis and babesiosis. The most frequent clinical signs detected in positive dogs for these CVBDs were: loss of weight, depression, lymph-nodes and spleen enlargement, conjuntivitis, keratitis, blepharitis, alopecia, ulcers/nodules, and exfoliative dermatitis. No other clinical signs were recorded.

Only two positive dogs for filarial infections (13.3%; 95% CI = 2.3–41.6%) showed clinical signs, but unspecific for clinical diagnosis of this CVBD. Both dogs showed loss of weight, whilst only one of these, positive also for leishmaniosis, had blepharitis, and conjunctivitis.

At physical examination, thelaziosis was not found in any of the tested dogs.

It should be noted that the 716 dogs were analyzed also for intestinal nematodes (*Trichuris, Toxocara, Toxascaris* and Ancylostomidae), cardiopulmonary nematodes (*Angiostrongylus* and *Capillaria*) and cestoda (*Dipylidium* and other Taeniidae) (unpublished data), but no significantly association was found between them and vector borne diseases investigated in this study (*P* > 0.05).

## Discussion

The limited extension of the Molise region territory, the small size of its dog population (56,729; www.lav.it) and the availability of an efficient veterinary system of active surveillance made it possible for us to have an accurate scenario of leishmaniosis and other CVBDs in dogs in this poorly investigated region.

Specifically, a prevalence of 10.2% of leishmaniosis, 2.1% for filarial infections and 0.3% for *B. canis canis* was found.

Canine leishmaniosis caused by *L. infantum* is endemic in all countries of the Mediterranean basin, but it is spreading northwards (20). In Italy, the median seroprevalence of leishmaniosis in 377 studies on dogs since 1971 to 2006 was 17.7% (range 10.7–21.1%), but prevalence of leishmaniosis is increasing over 40% in different zones not only of southern Italy, but also of northern Italy (12, 22, 23). In the present study, the prevalence recorded in Molise was 10.2%. Not many data are available about canine leishmaniosis in this region, but the more recent indicate an average prevalence of 18% in 2016 (unpublished data provided by CReNaL). The lower prevalence reported in our study can be correlated with enrolled dogs that were chosen *at random*, whilst the sera of dogs detected by CReNaL were all symptomatic for leishmaniosis.

This is the first time that *B. canis canis* is reported in the Molise region. PCR-positive prevalence (0.3%) for this parasite was higher than prevalence reported in hunting dogs from southern Italy (0.15%) (24) and from northern Italy (0%) (25, 26) but lower than a study conducted in central Italy with a prevalence of 2.3% (25). Higher prevalence values were reported in a study performed on 103 dogs from northern, 43 form central and 18 form southern Italy with PCR-positive prevalence of 29.1, 4.7, and 11.1% respectively, but all enrolled dogs in this study had clinicopathological findings compatible with tick-borne diseases (27).

Prevalence of filarial infections reported in this study (2.1%) was not very high. *A. reconditum* was the most prevalent (1.3%) filarial species and noteworthy was reported for the first time in the Molise region. The zoonotic *D. repens* was found in 0.8% of the tested dogs. Interestingly, a case of subcutaneous human filariosis caused by *D. repens* was described by Pampiglione et al. (28) in the arm of a children in Campobasso, one of the two provinces of the Molise region. In the same study the Knott's test was performed also on blood from 135 dogs, over 2 years old, confirming the presence of *D. repens* (3.0%) in dogs living few km from the clinical study (28). *D. immitis* was found in this study in 2 dogs (0.3%). For a long time *D. immitis* was recorded only in the Po River Valley, but more recently this parasite has spread to previously non-endemic areas of central and southern Italy (14, 29, 30). The two positive dogs were autochthonous hunting dogs that had never been abroad. A significantly higher association between hunting dogs and filarial infections was shown, according to literature (31, 32).

In this study, 3 dogs showed co-infections of *L. infantum* and *B. canis canis* (0.1%) or *A. reconditum* (0.3%). No associations were found between intestinal and cardio-pulmonary parasites and vector borne diseases investigated in this study. Recently, Baxiaras et al. (33) showed the severity of clinical signs of leishmaniosis is increased where a co-infection with other vector-borne pathogens is present. In some studies, a predisposition to leishmaniosis in dogs with other vector-borne pathogens has been described (34–36).

In conclusion, the detection of CVBDs in dogs with or without clinical signs (37) reinforces the importance of increasing the veterinary community, owners and public health authorities' awareness of the risk of infection, highlighting the need to plan effective control programs to guarantee the health and welfare of pets, and to enhance the safety of people (38).

## Data Availability

The datasets generated for this study are available on request to the corresponding author.

## Author Contributions

MG, VF, GC, GO, LR, and MPM contribute conception and design of the study. LC, MEM, and NE organized the database. MG, LR, and MPM wrote the manuscript. MEM and MPM performed the statistical analysis and GIS. All authors contributed to manuscript revision, read and approved the submitted version.

### Conflict of Interest Statement

The authors declare that the research was conducted in the absence of any commercial or financial relationships that could be construed as a potential conflict of interest.
